# Supplementary material for: Masticatory dysfunction in patients with diabetic neuropathy: A cross-sectional study
Source: PLoS One. 2022 Jun 6;17(6):e0269594. doi: 10.1371/journal.pone.0269594 (PMC9170089; doi:10.1371/journal.pone.0269594)
Supplement: Attachment — Submitted filename: Response to Reviewers.docx [file pone.0269594.s002.docx]

April 11, 2022

Dr. Kanhaiya Singh, Ph.D

Acadmice Editor

PLOS ONE

Dear Dr. Kanhaiya Singh and reviewers:

We are very grateful to have been given the opportunity to revise our manuscript “Masticatory dysfunction in patients with diabetic neuropathy: A cross-sectional study (ID: PONE-D-22-02324)”.

We appreciate the constructive and intuitive comments from the reviewers, which have resulted in significant overall improvement of the paper’s quality. Please see the specific answers to the reviewer’s insightful comments in a point-by-point fashion as below.

Response to Reviewer Comments

Reviewer #1

1. While the paper entitled “Masticatory dysfunction in patients with diabetic neuropathy” is interesting and insightful, some revisions of the text are needed.

Response: We are delighted to hear that the reviewer thinks our manuscript is interesting, albeit with some drawbacks. To increase the quality of our manuscript, we followed the suggestions described below.

2. Materials and Methods: since the paper focuses on diabetic neuropathy, the fact that it was exclusively diagnosed by means of clinical examination, without the use of any of the available quantitative tests, is problematic. Moreover, it precludes the possibility to investigate the association between the objective degree of neuropathy and the reduction in masticatory efficiency.

Response: We completely agree with this reviewer’s insightful criticism. In this present study, diabetic neuropathy was diagnosed using the abbreviated diagnostic criteria. These criteria are frequently employed for the diagnosis in Japan and are commended by Diabetic Neuropathy Study Group in Japan because of their sensitivity (68%) and specificity (74%) obtained by evaluating nerve conduction test as a gold standard (Yasuda H et al., Diabetes Res Clin Pract, 2007). Thus, we believe our present study using the abbreviated diagnostic criteria can support our conclusion. However, as the reviewer pointed out, the quantitative analysis such as the nerve conduction test should be helpful to assess the association between the objective degree of neuropathy and the reduction in masticatory efficiency. This point is discussed in the revised discussion section.

Materials and Methods section: Page 6, line 118-121

Discussion section: Page 14, line 285-289

3. Patient description is also problematic: a major bias is due to the fact that no mention is made of the occlusal condition of the patients the presence of prosthodontic rehabilitation, which is very likely to be present given the age and condition of the subjects, and which is certainly capable of altering masticatory efficiency. No information is given as to whether the masticatory efficiency test was carried out with or without removable prostheses in place. Moreover, the presence of fixed prosthodontic rehabilitation, either on natural teeth or implants, should be noted and taken into account. No attempt was made to stratify the subjects according to the number of teeth still present, even though it is well attested that masticatory efficiency sharply decreases if the number of remaining teeth decreases below 20. In the context of this research, a few subject with an extremely compromised dentition may have skewed the analysis exaggerating the effect of tooth loss. At the very least, the presence of prosthodontic rehabilitation should be discussed.

Response: We sincerely appreciate this reviewer’s keen eye. Prosthodontic rehabilitation and the number of remaining teeth should be considered in more detail. First of all, the removable protheses were used in 46 patients, whereas no dental implant was observed in all subjects. Besides, the measurement of masticatory efficiency was conducted with the removable prostheses in place. These points are described in the revised Materials and Methods and Results section.

In addition, as the reviewer pointed out, it is true that the reduced number of remaining teeth, less than 20, should affect oral function, including masticatory efficiency. Including such subjects may have skewed our analysis. Accordingly, encouraged by this reviewer’s constructive comment, we conducted a stratified analysis of the patients with more than 20 remaining teeth. As a result, we found that diabetic neuropathy decreased mastication efficacy. This finding can support our conclusion that the complication of diabetic neuropathy is associated with masticatory disfunction.

Materials and Methods section: Page 7, line 143-144

Page 7, line 158-162

Results section: Page 8, line 173-174

Page 11, line 223- Page 12, line 235

New Figure 3

New Table 3

Discussion section: Page 13, line 245-248

4. Results: the meaning of line 160-161 is unclear. Line 183-184 is unclear in the context as well, and needs to be clarified (positively or negatively associated?). the same goes for line 210 (…were associated with reduced masticatory efficiency).

Response: In accordance with the reviewer’s suggestion, we have amended these unreadable sentences.

Results section: Page 8, line 169-171

Page 10, line 195

Page 10, line 211-213

5. Discussion: in the context of this paper’s research, to hypothesize a role for diabetic neuropathy in masticatory dysfunction is not of itself implausible (although it should probably be quite advanced to manifest on the trigeminal nerve), but it really is not well tested in the context of this research. The only way to do it is to separate the confounding factor or remaining teeth (a bias in this case) and investigate the effects of neuropathy on neuromuscular control of mastication, not on masticatory efficiency. This distinction, that admittedly reduces the claim of the paper, should be included and discussed.

Response: We sincerely appreciate this insightful suggestion. We agree entirely that this present study is still enough to draw the precise association between diabetic neuropathy and masticatory function, even though a new stratified analysis can solve the remaining teeth number problems. Following the reviewer’s constructive comments, we will conduct the next study assessing the neuromuscular control of mastication. This point regarding the future study is discussed in the revised Discussion section.

Discussion section: Page 14, line 289-291

Reviewer #2

1. The manuscript is well written, the statistical data are satisfactory and self explanatory. The figures provided in the manuscript lacks visual clarity and can be made more prominent. There are some background correction to be made in each figure for clarity. Thanks.

Response: We sincerely appreciate these positive and encouraging comments on our manuscript. Following this reviewer’s constructive suggestion, we have amended the incorrect notation in Figure 1. Besides, Figures 1 and 2 are revised to be more visible.

Revised Figure1 and 2
