# Supplementary material for: Masticatory dysfunction in patients with diabetic neuropathy: A cross-sectional study
Source: PLoS One. 2022 Jun 6;17(6):e0269594. doi: 10.1371/journal.pone.0269594 (PMC9170089; doi:10.1371/journal.pone.0269594)
Supplement: Attachment — Submitted filename: Response to Reviewers.docx [file pone.0269594.s003.docx]

May 8, 2022

Dear Dr. Kanhaiya Singh, Ph.D

Academic Editor

PLOS ONE

We are particularly appreciative of your positive comments on our manuscript “Masticatory dysfunction in patients with diabetic neuropathy: A cross-sectional study (ID: PONE-D-22-02324R1)”.

Meanwhile, we have taken the opportunity to make the suggested revisions in the latest round of peer review. The changes made in response to comments from reviewer #1 have increased the quality of the paper. Please see below for our point-by-point responses to the critiques of reviewer 1.

Response to Reviewer Comments

Reviewer #1

1. The revised manuscript has been improved, but some points are still lacking. Considering the significant limitations of the masticatory ability testing system as methodology for the evaluation of a complex pathology such as diabetes in this study, this paper needs to be careful about the strength of the conclusions. Association does not imply a cause/effect relationship, and demonstrating one will require experimental evidence, which is, by its very design, not presented by the study. Nevertheless, it is useful and inherently interesting to speculate on the possible mechanism linking diabetes and reduced mastication, given the high prevalence of both conditions and the relevance of the topic.

Response: We appreciate this reviewer #1, who acknowledged that our first revision responding to the previous questions was improved and our study could be potentially interesting. Besides, we agree with the new critiques suggesting the effectiveness of the other examinations. To increase the quality of our manuscript, we followed such constructive suggestions described below.

2. Introduction: Please mention the actual possibility of recording the mandibular kinematic of the chewing patterns during chewing that gives information on the peripheral effects of neuromuscular motor control that would be useful for understanding the neuropathic effects of diabetes(doi: 10.1093/ejo/cjr109.).

Response: We completely agree that assessing the mandibular kinematic is helpful in clarifying the neuromuscular motor function. Thus, this point was mentioned in the revised introduction section with appropriate reference (Piancino et al., 2021, European Journal of Orthodontics).

Introduction section: Page 3 line 85-87

3. Discussion: Line 267 please add that to properly consider the role of sarcopenia during mastication it is necessary to record the mandibular kinematics and muscular activation during chewing (doi: 10.1016/j.archoralbio.2016.03.013.). To this end methods other than masticatory ability testing system are needed.

Please add at the end of the discussion as limitation of the study that the methodology used i.e. the ability testing system, is not refined enough for the study of a complex pathology such as diabetes and that the future direction will be the recording of the mandibular kinematic together with the muscular activation to establish a deeper understanding of the topic.

Response: We sincerely appreciate this insightful comment. Monitoring the mandibular kinematics and muscular activation should be helpful in clarifying the relationship between sarcopenia and masticatory function. Following this reviewer’s suggestion, we introduced this idea with a proper reference (Piancino et al., 2016, Archives of Oral Biology) in the revised manuscript. Besides, at the end of the discussion section, we have referred that the additional examinations recording mandibular kinematic and muscular activity will strengthen our findings in this present study.

Discussion section: Page 13, line 268- Page 14, line 271. Page 15, line 301-303

4. Abstract and Conclusion: Please, add a reference to the methodological limitation of the study.

Response: In accordance with the reviewer’s comment, we have referred to the necessity of the mandibular kinetics and muscle activity examinations in the revised conclusion section. On the other hand, the abstract section, having a character limit, simply referred to the results demonstrating that diabetic patients who developed diabetic neuropathy had reduced masticatory efficiency. Thus, we think the abstract in this paper may not need to be amended from its current form.

Conclusion section: Page 16, line 307-309.
